# Supplementary material for: Content-rich biological network constructed by mining PubMed abstracts
Source: BMC Bioinformatics. 2004 Oct 8;5:147. doi: 10.1186/1471-2105-5-147 (PMC528731; doi:10.1186/1471-2105-5-147)
Supplement: Additional File 5 — The original Chilibot query results of the term "long-term potentiation (LTP)" and 22 other terms, limiting the latest references analyzed to the years 1990, 1995, 2000, and 2004. [file 1471-2105-5-147-S5.bz2 › chilibotAdditionalFile5/ltp2004/html/TRKA_TRKB.html]

 


 **TRKA** and **TRKB** 
  
Found 393 abstracts in PubMed,  **30 abstracts were retrieved and analyzed**.  


---

 Search Google  |
 PDF files only 
|  EDU domain only 

---

**Interactive relationship** (e.g. stimulation, inhibition, etc)

- Depletion of target derived  **trkB**  ligands by injection of  **trkB**  Fc fusion protein into the superior colliculus increases the peak rate of RGC death compared with  **trkA**  Fc treated and normal animals.  Ref: 14602830 J Neurosci, 2003
- Rat and mouse cochleae were immunolabeled with a rabbit anti Trk A  [ **TRKA** ]  polyclonal antibody Chemicon that has no reported cross reactivity with Trk B  [ **TRKB** ]  and Trk C.  Ref: 14698082 Hear Res, 2004
- Trk A  [ **TRKA** ]  was not observed in stria vascularis, hair cell stereocilia, nor in the Trk B  [ **TRKB** ]  and Trk C rich cerebellum.  Ref: 14698082 Hear Res, 2004

**Parallel relationship** (e.g. studied together, co-existance, homology, etc.)

- Tyrosine kinase receptors, including Trk A  [ **TRKA** ] , Trk B  [ **TRKB** ]  and Trk C, participate in many different biological processes that are regulated by neurotrophic factors.  Ref: 14698082 Hear Res, 2004
- Expression of the neurotrophin receptors Trk A  [ **TRKA** ]  and Trk B  [ **TRKB** ]  in adult human astrocytoma and glioblastoma.  Ref: 12711810 J Biosci, 2003
- The mRNA and protein levels of the neurotrophins of the NGF family NGF, BDNF, NT 3 and NT 4 5 and their receptors  **trkA** ,  **trkB** , trkC and p75 were quantified in septum and hippocampus using RT PCR.  Ref: 14557907 Exp Brain ResExp Brain Res, 2003
- With this end in view, the present study has examined 10 tumour biopsy samples identified as astrocytoma, pilocytic astrocytoma and glioblastoma and peritumoral brain tissue of adult patients, for the presence of Trk A  [ **TRKA** ]  and Trk B  [ **TRKB** ]  receptors, by immunohistochemistry.  Ref: 12711810 J Biosci, 2003
- It is hoped that the quest for molecular markers such as neurotrophin,  **TrkA** , and  **TrkB**  will continue to advance the understanding of neuroblastoma.  Ref: 14680322 Urol Clin North Am, 2003
- **TrkA**  immunoreactivity was visible in the nerve fiber layer,  **TrkB**  staining was within RGC bodies, TrkC was undetectable, and p75 was widely expressed across the retina, within the Müller glia.  Ref: 14507902 Invest Ophthalmol Vis Sci, 2003
- About 3 0, and 20% of the lumbar DRG neurons expressed  **trkA** ,  **trkB**  and trkC mRNAs, respectively.  Ref: 12573533 Brain Res Mol Brain Res, 2003
- The presence of neurotrophins and the neurotrophin receptors p75 p75NTR, tyrosine kinase A  **TrkA** ,  **TrkB**  and TrkC have been described in several immune cells.  Ref: 14699973 Prog Brain Res, 2004
- Results of RT PCR indicate that P19 neurons express several neurotrophin receptors,  **trkB** , and trkC, but not  **trkA**  but they do not express any of the four neurotrophins.  Ref: 12672017 J Neurobiol, 2003
- **TrkB**  and  **TrkA**  like immunoreactivity was never found.  Ref: 14758546 Anat Embryol (Berl)Anat Embryol (Berl), 2003
- ...  **TrkA** ,  **TrkB** , and TrkC, were localized in myoepithelial cell and ductal cell membranes.  Ref: 12882781 Invest Ophthalmol Vis Sci, 2003
- In this study we investigated the presence and cell localization of Trks  **TrkA** ,  **TrkB**  and TrkC in taste buds of the zebrafish Danio rerio using Western blot and immunohistochemistry.  Ref: 14700728 Neurosci LettNeurosci Lett, 2003
- Although neurotrophins did not regulate p75NTR processing, the alpha and gamma secretase mediated cleavage of p75 is modulated by receptor tyrosine kinases Trks  **TrkA**  and  **TrkB**  but not TrkC.  Ref: 12843241 J Neurosci, 2003
- Both  **TrkA**  and  **TrkB**  immunostained cells were widely distributed in the lateral, medial and spinal vestibular nuclei, and were less frequently seen in the superior vestibular nucleus, x and y subnuclei.  Ref: 12858046 Neuroreport, 2003
- Cells from the immune system have been detected in CNS injury and found to produce and secrete a variety of neurotrophins such as NGF, BDNF, NT 3 and NT 4 5, and to express similarly to neuronal cells, members of the tyrosine kinase Trk receptor family such as  **TrkA** ,  **TrkB**  and TrkC.  Ref: 14699975 Prog Brain Res, 2004
- To explore this apparent regional difference in BDNF action, we compared expression of the neurotrophin receptors  **trkA** ,  **trkB**  and trkC.  Ref: 12966210 Dev NeurosciDev Neurosci, 2004
- Messenger RNA signals for c ret, a common signaling receptor of GDNF family ligands, were seen in about 60% of DRG neurons, and some of these neurons expressed  **trkA** ,  **trkB** , or trkC mRNAs.  Ref: 12573533 Brain Res Mol Brain Res, 2003
- The distribution of nerve growth factor NGF, brain derived neurotrophic factor BDNF, neurotrophin 3 NT 3 and 4 NT 4, and their high affinity receptors  **TrkA** ,  **TrkB** , TrkC and low affinity receptor p75, was analyzed in adult porcine retinal sections by immunohistochemistry.  Ref: 14507902 Invest Ophthalmol Vis Sci, 2003
- Anti  **trkB**  antibodies also reduced cAMP response element binding protein phosphorylation in the hlDBB and combined injections of anti  **trkA**  and  **trkB**  in the olfactory bulb reduced estrogen induced increases in basal forebrain choline acetyltransferase.  Ref: 12960034 Endocrinology, 2003
- In agreement with these results the sensory cells of taste buds, displayed  **TrkA**  and  **TrkB**  like... immunoreactivity.  Ref: 14700728 Neurosci LettNeurosci Lett, 2003
- the high affinity receptors  **TrkA** ,  **TrkB** , and TrkC.  Ref: 12915691 J Clin Endocrinol Metab, 2003
- The trk receptor antibodies, tested by Western blot on human cerebellum homogenates, revealed multiple immunoreactive bands for  **trkA**  and single bands for  **trkB**  and trkC.  Ref: 12927579 Int J Dev Neurosci, 2003
- Both flat and process bearing ECs were immunopositive for brain derived neurotrophic factor, nerve growth factor, neurotrophin 4, and  **TrkB**  but not  **TrkA** .  Ref: 12555206 Glia, 2003
- **TrkA**  and  **TrkB**  co existed in the same taste buds, but remains to be clarified whether or not they are co expressed in the same cells.  Ref: 14700728 Neurosci LettNeurosci Lett, 2003
- To determine which of the neurotrophins NTs nerve growth factor NGF, brain derived neurotrophic factor BDNF, neurotrophin 3 NT3, and neurotrophin 4 5 NT4 and their receptors NTrs,  **TrkA** ,  **TrkB** , TrkC, and p75, are present in the adult rat lacrimal gland.  Ref: 12882781 Invest Ophthalmol Vis Sci, 2003
- Phosphorylation assay of various Trk receptors overexpressed in fibroblasts suggested that NT 7 could activate  **TrkA**  but not  **TrkB**  or TrkC.  Ref: 12546061 Mol Cell Neurosci, 1998
- To elucidate the underlying mechanisms expression of the neurotrophin receptors p75NTR,  **trkA** ,  **trkB** , and trkC on eosinophils was analyzed by RT PCR and immunocytology.  Ref: 12900521 J Exp Med, 2003
- In this work the expression and anatomical localization of NGF, BDNF and NT 3 and of corresponding high affinity receptors  **TrkA** ,  **TrkB**  full length and truncated TR isoforms, TrkC and of the low affinity p75 receptor were assessed in surgical samples from adult human lung by reverse transcriptase polymerase chain reaction RT PCR, Western blot and immunohistochemistry.  Ref: 12791675 Am J Respir Cell Mol Biol< MedlineTA>Am J Respir Cell Mol Biol, 2003
- These findings suggest that Trk A  [ **TRKA** ]  and Trk B  [ **TRKB** ]  are involved in tumour pathogenesis, especially in the early stage, and may respond to signals that elicit glial proliferation, and thus contribute to progression towards malignancy.  Ref: 12711810 J Biosci, 2003
- RT PCR showed positive bands at the appropriate sizes for NGF, BDNF, NT3, and NT4, and for the receptors  **TrkA** ,  **TrkB** , TrkC, and p75.  Ref: 12882781 Invest Ophthalmol Vis Sci, 2003
- Proteins of 140 and 145 kDa, identified as full length  **TrkA**  and  **TrkB** , were detected.  Ref: 14700728 Neurosci LettNeurosci Lett, 2003
- In the peritumoral brain tissue, only neurons showed IR for Trk A  [ **TRKA** ]  and Trk B  [ **TRKB** ] .  Ref: 12711810 J Biosci, 2003
- The majority of rat nodose neurons express  **TrkA** ,  **TrkB**  and TrkC receptor after 1 day in culture.  Ref: 12493590 Neurosci Lett, 2003
- TNF in medium sized neurons colocalized with neurofilament and  **trkB** ...  **trkA**  or RET, suggesting a selective phenotypic switch in presumably low threshold myelinated primary afferents.  Ref: 12603269 Eur J Neurosci, 2003
- Particularly, the gene expression of NGF, NT 3, and the high affinity receptors  **trkA** ,  **trkB**  and trkC increased significantly.  Ref: 14557907 Exp Brain ResExp Brain Res, 2003
- Consistent with the presence of  **trkB**  but not  **trkA** , BDNF causes rapid phosphorylation of MAP kinases ERK1 and ERK2, but NGF does not.  Ref: 12672017 J Neurobiol, 2003
- Trk A  [ **TRKA** ] , Trk B  [ **TRKB** ]  and Trk C expression has been reported in the rat ventral cochlear nucleus.  Ref: 14698082 Hear Res, 2004
- The immunohistochemical occurrence of the high affinity neurotrophin NT receptors  **trkA** ,  **trkB** , and trkC is shown in the pre term newborn, infant, and adult human post mortem cerebellum.  Ref: 12927579 Int J Dev Neurosci, 2003
- Western blot analysis confirmed these results, showing that the lacrimal gland expresses NGF, BDNF, NT3, and NT4 as well as the NTrs  **TrkA** ,  **TrkB** , and TrkC and the p75 protein.  Ref: 12882781 Invest Ophthalmol Vis Sci, 2003
- We show here that their respective receptors,  **TrkA** ,  **TrkB** , TrkC, as well as p75 NTR the low affinity NGF receptor, are expressed in vitro by the nasal ensheathing cells.  Ref: 14730705 Glia, 2004
